# Medical Societies and Clinical Reports

**Published:** 1858-03

**Authors:** 


From the Medical and Surgical Reporter.

### *Medical Societies and Clinical Reports.—Philadelphia County Medical Society.*

*Rheumatism.*—Jan. 13th, 1858. Dr. Emerson in the chair. The subject for discussion was rheumatism, on which Dr. R. K. Smith read the following paper. In presenting the subject of acute rheumatism, for the consideration of the society, he did not propose views that have not already received the careful attention of many eminent medical philosophers. There are, however, notwithstanding the varied investigations of the causes, pathology, and treatment of this disease—few medical writers who agree upon either. This diversity of opinion, in regard to one of the most common affections, the physician is called upon to treat—a disease, too, which often involves the very citadel of life, and always entails upon the patient an amount of suffering and helplessness that enlists the warmest sympathy—demands a scrutiny so thorough and searching as to lead to greater harmony in regard to its pathology, and more uniformity in its treatment.

With a disposition to elicit discussion upon some of the unsettled points to which I refer, the following interrogatories are proposed, touching the cause, pathology, and treatment of rheumatic fever.

1st. Is the primary cause of acute rheumatism a morbid poison in the blood, or, is it located in the nervous system.

2d. If in the blood, is it *urea*, or what other poisonous matter?

3d. How can pericarditis, and endocarditis, as consequences of rheumatism, be explained upon the theory that it is a nervous disease?

4th. Are the consequences ultimately fatal?

5th. What tissues are invaded by rhenmatic inflammation, and does it ever terminate in suppuration or gangrene?

6th. Do not the remedies found most serviceable in rheumatism, exercise more influence upon a blood poison than upon the nervous system?

7th. Is a strict antiphlogistic treatment proper in an ordinarily robust patient?

8th. What value is attached to the treatment with lemon-juice?

9th. What are the merits of the alkaline treatment?

10th. Of the calomel and opium treatment?

11th. How is the action of colchicum explained, and what is its value as a remedy in rheumatism?

12th Are local applications beneficial, and, if so, what are to be used?

In suggesting these questions, it is proper for me to state the principles which govern me, in the treatment of rheumatism, and to briefly reply to my own questions.

In regard to the first query—the primary cause of acute rheumatism—I hold the doctrine that some morbific agent contained within the blood is the original cause of the disease. That it is this deterioration that constitutes what has been called the "rheumatic dyscrasia." That some pre-existing change in assimilation and excretion renders the system susceptible to the exciting causes of rheumatism; poisonous matter of a peuliar nature is deposited within the blood, and the disease is developed. The evidences adduced, are the absolute changes known to exist; the excess of fibrin, and the alteration in its chemical composition.

To the second question, I answer: Urea is found in the blood of patients laboring under acute rheumatism, and that certain agencies employed for the relief of the diease, eliminate it from the blood by increasing it in the urine. I, however, do not contend for the point that *urea* is the *only* poisonous matter in the blood, that generates arthritic diseases.

The third question, I leave to gentlemen who belive in the nervous origin of rheumatism, to answer. Upon such a theory, I can understand no explanation of the causes of pericarditis and endocarditis, compatible with reason and physiology. Such men as Fothergill, Willan, Morton, McIntosh, and Haygarth, have failed to show the connection, and I shall not attempt it, especially as I do not believe the doctrine.

In reply to question fourth, I believe that pericarditis and endocarditis eventually terminate in death. Not that an attack may not be relieved, in fact, *supposed* to be cured, but that certain structural alterations of the heart occur, which are never entirely removed, and will ultimately terminate life.

The tissues involved in the inflammation of rheumatism, are the fibrous and fibro-serous, and whenever other tissues are invaded, it is in consequence of their near contiguity to the original disease. This disease, I believe, when unattended by any complication, never terminates in suppuration or gangrene.

6th. In regard to the "*modus operandi*" of the remedies which relieve; in rheumatism; this involves the question as to which theory is correct whether it is a disease of the nervous system or of the blood. Adopting the latter view, I can conceive the action of certain substances upon the fluids of the system so as to eliminate or neutralize certain other elements; and I leave to the members the application of their own experience in explanation of the method by which cures are effected through the interposition of the agents they employ.

7th. That the antiphlogistic treatment of rheumatism, followed out in its most rigid sense, has been successful, I know, but that it is the proper course to pursue, in treating this inflammation, I deny. The inflammation and fever which attend rheumatism differ widely from ordinary inflammation; they have more a character of irritation, and present various phenomena unknown in any other inflammatory fever. The state of the skin is one of these peculiarities, and would suggest to every thoughtful man, the propriety of withholding every means that would stimulate the perspiratory organs to further action. Bleeding is unquestionably an important remedy in many cases, and its utility does not oppose the theory that a blood poison produces the disease.

8th. The use of *lemon juice*, in acute rheumatism, according to Dr. Babington, has been eminently successful. Indeed, I may say that, in his hands, its curative effects were astonishing. It will, no doubt, surprise most physicians, to be told that, in a large number of cases treated in this way, the disease was thoroughly cured in from *three* to *eight days*, and without the employment of any other means, whatever, except an occasional dose of the comp. pulv. rhei, when the bowels required it. The doses directed by Dr. B. were from three to six ounces, three times a day, without admixture of any kind. The reason that others have not been so successful with this remedy, he tells us, is because they do not administer it in sufficiently large doses. Dr. Dalrymple unites, also, in ascribing to the lemon-juice extraordinary curative powers, which, if true, there is a value in the remedy which should not be overlooked.—

How it acts has not been explained, but why it should not possess a power to eliminate the poisonous matter from the blood, equal to other remedies, or even in a superior degree, I cannot understand. My own experience in the use of lemon-juice in rheumatism is limited, and, in these large doses, I have had none.

Its efficacy in my hands did not meet my expectations, but this may have been in consequence of the smallness of the dose.

9th. The use of *alkalies*, for the cure of rheumatism, is just now the popular treatment; whether they deserve the first place among the remedies is for experience to determine. They are used chiefly in the form liq. potass. and potass. bicarb., with a solution of the latter in the proportion of ℥j to aqua Oj, as a local application. The question will, no doubt, give us the benefit of our fellow-members' experience. In eliminating or neutralizing the poison, upon which the inflammatory action depends, there appears to be no incompatibility in the harmonious action of such opposite remedies as lemon-juice and liq. potassæ, unless this action be fully explained to be so.

10th. Calomel and opium in rheumatism is a hackneyed treatment which we have all practiced with varied success. The patients have recovered, and we have not pushed the remedies so far as to do them injury. At best, the merits of this course appear to be negative.

11th. Colchicum has been one of my favorite remedies in this disease, but I am free to say that it has frequently disappointed me in the effect anticipated, and never produced the results in my hands which have been attributed to the alkalies and lemon-juice. From some expeirments published by Dr. Maclagan, of Edinburgh, the physiological action of colchicum in diminishing urea from the blood, and passing it off by the urine has been conclusively settled. Whether its beneficial effects are produced by such action is yet uncertain.

12. In regard to local applications in acute rheumatism, my dependence is solely upon leeches and ice, or iced water; the ice or iced water I consider invaluable, and have used it in very many cases, and *never* without the most decided benefit, both as regards the comfort of the patient and the curtailment of the disease. I have repeatedly pursued the inflammation from joint to joint, and from one side of the body to the other in its fugitive course, and for the first time have to see a case of metastais to the heart, where they have been used. I do not belive this result can be brought about by such treatment, *i. e.* that it is productive of it. It may occur under the treatment, but, as I have seen it repeatedly where this remedy had not been resorted to, it would be strange, indeed, if rheumatism should never metastate where cold applications were used. There is in my judgment unnecessary alarm manifested in regard to the danger of the remedy.

Dr. REMINGTON belived all the tissues were affected by it. He believes that he had seen it the brain, stomach, and bowels. He had seen a serious case of rheumatism of the bowels some years ago, which recovered after a long indisposition extending over three months. The case eight or ten years afterwards, died under quack treatment very suddenly, and with every appearance of a metastasis to the heart, under the use of cold sheets and packing. He also mentioned the case of a girl 12 or 14 years old, who took a bath and then studied her lesson under an open window. He used vin. colch. rad. combined with an anodyne, as morph. sulph., aq. camph., and spt. æth. nit, perhaps the iodide of potassium, or the nitrate, if fever presented. The case recovered rapidly. Along with this he gave a pill of comp. ext. colocynth, acet. ext. colchicum and calomel. After five or six visits she was restored. Nitrate of potass, was very efficacious. Tartrate of antimony, especially in the articular form, he did not like. Lemon-juice he did not rate very high. If there was gout with it, this would certainly do a great deal of mischief. Bleeding was of use sometimes; cupping very advantageous. *Ice* was *never beneficial.* We often see it affecting the heart, and often proves fatal in these cases, hence we should prefer to have it externally rather than internally. In general, physicians, especially the juniors, are too apt to think they do everything and nature nothing. Now he thought the reverse; the *vis medicatrix naturæ* should not be overlooked.

Dr. Bell thought the lecturer was rather brief. A very important thing is to impress on the public mind the risk of its becoming rheumatism of the heart of a grave kind. This is not so fatal as is thought. The sero-fibrous and muscular, were the tissues affected. Shall it be regarded as a blood disease or not? Every disease was to a certain extent a disease of the blood. If we prevent the skin from acting, as where animals have been sealed up as it were, in various experiments, we produce a disease of the blood. The common treatment of venesection is very valuable and important, especially if the heart is attacked. A fact interesting to know, is the proportion of cases of rheumatic inflammation of the articulations to those of the heart. In 136 cases of rheumatism, the heart was affected in 90, as one writer tells us. Dr. B. was better pleased with bloodletting in endocarditis and pericarditis than in other cases. He had treated a case in a young man for another physician with partial relief, when he had a return of it in the heart. He then bled

*frequently* and observed as a result, in a subject who was liable to repeated attacks previously, an entire immunity for years after. In more doubtful cases in which stimulants had been used freely, he should not hesitate to bleed, and had in such seen good effect as before, and a freedom from attacks afterwards. The *cure* of the attack occupies us too much to the exclusion of *prevention*. Sometimes he began by a more moderate course, as with colchicum and alkalies, antimony and opium, with relief and an apparent removal of the disease; but it returned: then he had recourse to depletion, as cups or leeches, and not always from the arm in a broken constitution, as from intemperance, &c.

This is not called for in subacute cases, and especially of pericarditis; we may then treat by opium and calomel, and it yields rapidly. In the chronic form with carditis, we have the best effect from iodine, as iodide of potassium, never in full doses, 5 grs. twice a day, with syr. sarsap. comp., or syr. of wild cherry. A far more troublesome form is the subacute, where it seems to get well, patient going about, and has a return of the disease, again laid up with excessive pain night after night. Opium fails to give relief, depletion does not do from the condition of the patient, he then becomes hopeless of relief and quackery is invoked.—Here the skin pours out an acrid sweat. In such cases (though it may seem homœopathic) diaphoretics are of service, but though like to like, yet being in sensible doses it cannot be termed that practice; pulv. ipecac. et opii, or opium with tartar emetic, and the vapor bath comes in with good effect. With a febrile condition, a shower bath, and even cold locally are beneficial. This is best in the acute form of the diseaes, and not when the skin has lost its power, and we have general weakness. He could say little in regard to the use of a particular remedy. He never used persistently one remedy, but varied them according to circumstances, colchicum, bleeding, purgatives, or opium. He had used lemon juice and watched closely its effects, but failed to derive such benefit as has been remarked by others.

Dr. Smith said, in regard to his paper, that he had no opportunity to make it more elaborate, having been very busy. His object was to present only prominent *points* on the subject, so as to elicit discussion. It especially relates to acute rheumatism, accompanied with high fever; the inflammatory form.

Dr. Turnbull asked Dr. S. to inform him what treatment he would make use of in a child taken with a chill, vomiting, fever, pulse 120, respiration oppressed, pain in the cardiac region, roughness of the sounds of the heart, and a murmur above the nipple? What course he would pursue if not using depletion and calomel? He had seen pericarditis and endocarditis relieved, and the patient to live some time after. High European authority was in favor of a cure of these affections, and with this treatment. These diseases are on the increase, and he would be sorry to think them utterly incurable. He had seen a case of disease of the heart, developed by influenza, and had seen it subdued by calomel, and subsequently iodide of potassium, in 5 grain doses, and now, three years after, she was entirely well.

Dr. SMITH thought he was misunderstood; he did not mean to propagate the opinion that pericarditis and endocarditis could not be relieved; they could be relieved, and were supposed to be cured; but when there was inflammation of the pericardium, or of the serous membrane lining the heart, he never saw a patient so radically cured that the patient would not die eventually from heart disease, unless cut off accidentally. Cases of heart disease, dying suddenly, &c., when traced back, would be found to have had rheumatism united with an affection of the heart. Not one in ten, that had fallen from heart disease, but had pericarditis or endocarditis, and had been living on in apparent health. In regard to the case of Dr. Turnbull, he would pursue this course: leeches over the heart and meet symptoms in a rational way, as they presented.

Dr. BELL remarked that such cases were carried for years, and hence they should be treated to prevent extensive disease of the valves, which was a common result of neglected endocarditis, but not at all, if treated in a proper manner.

Dr. H. HARTSHORNE believed they were sometimes cured, living at least for many years without any sign of disease. We must not forget, however, the number of fatal cases of heart disease which are not inflammatory in their origin. The local stiffening of the muscles, alluded to by Dr. Bell, and so common as an effect of partial exposure to the cold, he would class under simple inflammation, making a different disease of true articular rheumatism, or rheumatic fever. Many families never have genuine rheumatic fever, or acute articular rheumatism. Whether urea is the poison is very doubtful. When we can trace the effects of urea, retained in the blood, we do not have symptoms of articular rheumatism. The suggestion that it may be lactic acid, in excess, is more free from positive objection, but we know very little of the effect of lactic acid in the blood. Care is certainly needed in the use of cold water to the surface in rheumatism. Dr. H. believed it to be more hazardous when the fever is high than when subdued and more local, as then the poison may be supposed to have been in part eliminated, and the danger of metastasis less. Even the repulsion of the blood from the surface may be, in ordinary cases, a cause of internal inflammation.

Dr. SMITH said that Dr. Maclagan, in his experiments, found urea in the blood, and then, under the effect of medicine, it left the blood, and was found in the urine. These facts seemed very conclusve to this point. He would ask the gentleman to explain how cold does repel the blood, &c., and produce metastasis. Experience dose not prove it in acute rheumatism. He would like the *modus operandi* of the process, something more than mere prejudice against the remedy.

Dr. HARTSHORNE. Finding urea in the blood does not prove it the cause of rheumatism. Eminent physicians have observed the same thing in other diseases, as typhoid fever. The explanation of metastasis appeared to him very simple, if we take for granted that rheumatism is a disease of the blood. Whatever repels the blood from the surface tends to promote the deposit of morbid material in interior organs. When we pursue the diease from "joint to joint," this is a metastasis occurring under our eyes.

Dr. EMERSON (having called Dr. REMINGTON, Vice-President, to the chair). Rheumatism depends on different causes. The cold applications he considered, from his own experience, as fraught with danger. He should never forget one case, a stout man, with violent rheumatism of the lower limbs, and especially of the knee-joint. He was under treatment some weeks, and much improved, when Dr. E. had to leave the city, and made arrangements to send a friend, if necessary. The friends of the patient persuaded him to send for a hydropath, who subjected him to packing. The chest became painfully affected, and grew worse with every additional packing; still the hydropath stupidly persevered, and when Dr. E. returned, the wife had just interfered. He found him very bad, so that the least motion, or long breath, caused him to cry out. He was in a very critical condition. Dr. E. restored the disease to its former locality, by warm applications over the joint and lower limbs, and by the pulv. Doveri, and warm draughts, the pain left the heart and chest. In this case, there was every reason to believe that it left a locality where it was not dangerous for a vital position. He wished to call the attention of the members to a remedy which, in one case, produced a sudden and agreeble result: croton oil to the spine and back. It was in the case of a yong man, 19 years of age, with rheumatism in an aggravated form. The oil was applied freely, and getting on the shirt, spread over the back, and produced a most awful condition, and loud complaints fron the patient. He got well, and that faster than any cases Dr. E. had ever seen. He frequently applied it, and with very beneficial result. In regard to attacks of rheumatism in the heart, he knew no treatment so effectual as nitre ʒij, tartar emetic gr. j, calomel gr. j, and opium gr. j, divided into 12 powders, one taken three times a day. In corpulent women, as cooks, we have often complaints of pains at the heart and chest, which are traceable to rheumatic attacks; this treatment, sometimes alternated with, or succeeded by, potassii iodidi, was eminently successful.

Dr. NEBINGER thought he had seen two cases, in which by cold, metastasis was produced from the part affected to the heart. One was a young lady who was out in the evening to a party; shortly after she returned she was seized with violent pain in the ankle. Her mother, attributing it to a pain produced by dancing, poured cold water over it. This produced speedy relief, but immediately after she was seized with violent pain in the chest, oppressed breathing, &c. On being sent for, Dr. N. found, on examination, no reason for a belief in its being a sprain and diagnosticated rheumatism which had undergone a metastasis to the heart. He put the foot in hot water, and after mustard plasters, &c., it returned to its first locality and showed a case of acute rheumatism, by which several joints became affected. Here it was clear, cold water was detrimental. Another case was that of a man who had several times inflammatory rheumatism. On one occasion, after having suffered for several days, by the advice of a friend, he applied ice water and salt to the part. Although he experienced immediate relief in the part, yet violent pains set in in the cardiac region. He was bled freely, and before the bleeding was finished the pain was subdued. Sinapisms and other stimulants were applied to the joints, and after the disease was reproduced there, he was cured by the usual means. Having a predisposition to travel, is it good treatment to encourage it to do so by cold water? Some cases require very little, and others an immense amount of treatment. Of all remedies the "*hackneyed* remedy" is the best and hence he did not like this appellation. He did not like to hear good things called by bad names. Would we apply it to quinine, because so much used in autumnal diseases? Good remedies are solution of nitrate of potassa, colchicum, tartar emetic and opium. In the first severe case he ever saw of articular rheumatism, Dr. N. used cotton to the parts, and nitrate of potassa, tartar emetic and opium; it went on with little or no improvement for several weeks, and he changed the remedies; he at that time feared the use of mercury, feared the production of salivation; by and by, the case not improving, he determined to run all risks and used blue pill, with nitrate of potassa and opium, and in a little time it was cured. He reasoned thus: we have a patient sick a long time, several weeks suffering a great deal, and when grown quite dissatisfied with the treatment, and hope begins to quit us, we have recourse to this Sampson. Why delay? Why make use of the little Davids? Why not call upon Sampson at once? Since the time referred to, he had used mercury in every case of any severity without delay. He emptied the bowels, used the lancet if necessary, and then proceeded as follows ℞. Potassæ nitratis gr. x; Mass. pil. hydrarg. gr. j–jss; Ant. et. pot. tart. gr. $\frac{1}{12}$; M. Give one every 2 hours. Under this he had seen cases get well in ten or twelve days, when under other treatment they would have lasted weeks. He applied cotton to the joints. This was seemingly applying heat to a part already too hot. There is a certain amount of insensible perspiration going on, which collects between the cotton and the part to which it is applied, and this collecting it, acts as a local vapor bath; benefit was thus produced, and this, also, was an argument against the use of cold to the part.

Dr. BELL thought the difference in the reports of the experience of the gentleman may result from the different stages' constitutions, &c.— It is a vulgar error that the application of cold drives the blood into the internal organs. The heart responds to the application of cold to the surface, and we have a synchronous depression of its action.

Dr. HARTSHORNE would still have to remain in the *vulgar opinion* in common with all other physiologists except Dr. Bell. He could not consider the application of cold to the part a safe treatment in acute rheumatism.

Dr. SMITH thought Dr. H. would make the application of cold drive a little *more* poison to the heart. Now Dr. S. contended if there was any poison in the blood it was already in the heart, and that organ was as likely to take on the diseased action from the presence of an ounce as of a gallon of diseased blood. His theory was, that it was not the *amount* of poison that produced the effect. Dr. H. makes a certain amount of poison necessary to derange the organ, if his theory was correct. In regard to the term *hackneyed*, to which so much objection had been taken by Dr. Nebinger, he would say, that he meant to apply to the medicines calomel and opium no opprobrious epithet. On the contrary, there were no articles in the whole Materia Medica he more commonly used in general practice. He simply meant to speak of those remedies as being in almost universal use, and in the hands of the whole profession.

On motion, the society adjourned.

